# Roles of transcriptional factor 7 in production of inflammatory factors for lung diseases

**DOI:** 10.1186/s12967-015-0617-7

**Published:** 2015-08-20

**Authors:** Yichun Zhu, William Wang, Xiangdong Wang

**Affiliations:** Shanghai Respiratory Research Institute, Zhongshan Hospital, Fudan University Center for Clinical Bioinformatics, Fenglin Rd 180, Shanghai, 200032 China

**Keywords:** Lung disease, TCF7, TCF-1, Signal pathways

## Abstract

Lung disease is the major cause of death and hospitalization worldwide. Transcription factors such as transcription factor 7 (TCF7) are involved in the pathogenesis of lung diseases. TCF7 is important for T cell development and differentiation, embryonic development, or tumorogenesis. Multiple TCF7 isoforms can be characterized by the full-length isoform (FL-TCF7) as a transcription activator, or dominant negative isoform (dn-TCF7) as a transcription repressor. TCF7 interacts with multiple proteins or target genes and participates in several signal pathways critical for lung diseases. TCF7 is involved in pulmonary infection, allergy or asthma through promoting T cells differentiating to Th2 or memory T cells. TCF7 also works in tissue repair and remodeling after acute lung injury. The dual roles of TCF7 in lung cancers were discussed and it is associated with the cellular proliferation, invasion or metastasis. Thus, TCF7 plays critical roles in lung diseases and should be considered as a new therapeutic target.

## Background

Lung diseases are significant causes of death, of which chronic obstructive pulmonary disease (COPD), lower respiratory infection, or lung cancers are, respectively, ranked the number 3, 4, or 5th as the top 10 causes of death in 2012 globally, with the number of deaths as 3.1 million, 3.1 million and 1.6 million respectively [1]. In China, lung diseases are the fourth cause of death in 2012, with death rate at 75.59/100,000 in urban area, and 103.9/100,000 in rural area, and became the top one reason for hospitalization. The first cause of death in China is the malignant neoplasm, among which lung cancer is the most common one, causing a total death rate of 49.73/100,000 in urban area and 38.78/100,000 in rural area [2]. There are many types of lung diseases including obstructive lung diseases, infectious illnesses, lung cancer, respiratory failure, pulmonary edema, pulmonary embolism, pulmonary fibrosis and sarcoidosis, as well as occupational diseases.

Transcription factors as nuclear proteins bind to specific DNA sequences and regulate the transcription process of the gene, consisting of the largest family of human proteins encoded by about 8% of genes in human genome [3]. The specific DNA sequences binding to the transcription factors become the enhancer or promoter regions of the genes regulated. Transcription factors play the role in the regulation of the gene transcription through stabilizing or blocking the recruitment of RNA polymerase to specific genes, the catalysis of the acetylation or deacetylation of histone proteins, and the recruition of coactivator or corepressor proteins to the transcription factor DNA complex [4–6]. Transcription factors are critical for a huge number of cellular processes such as growth, development, and differentiation by regulating the gene expression [7]. Mutations of transcription factors were found in human diseases, and were considered as drugable targets for drug discovery and development [8, 9].

Transcription factor 7 (TCF7), also known as T-cell-specific transcription factor-1 (TCF-1), is a member of transcription factors. The recent paper overviewed the biology and functions of transcription factor 7 (TCF7), its interactions with other proteins in several critical pathways, and potential roles in lung diseases. We will explore the important role of TCF7 in pathogenesis, diagnosis, or therapies for lung diseases.

## Review

### Understanding the dual biological functions of TCF7

#### Structure and location

**Fig. 1:**
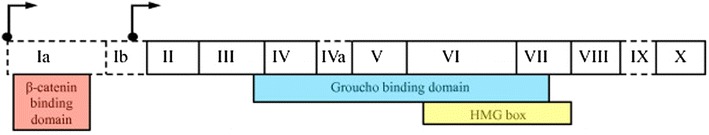
TCF7 gene structure. TCF7 gene contains multiple exons. Different splicing may transcribe to variants of mRNAs, and translates to different protein isoforms. Exon Ia, Ib, IVa, and IX are alternative exons. There exits two promoters as shown in the figure. The first promoter can generate mRNAs encoding a full-length activating form of protein isoforms which contains the β-catenin binding domain, while the second promoter cannot and thus produce the truncated dominant-negative isoforms. Therefore, two different groups of TCF7 isoforms function differently.

TCF7 gene was firstly identified as a T lymphocyte-specific transcription factor in 1991, and belongs to a large DNA binding protein family called high-mobility group (HMG) box [10, 11]. The TCF7 gene resides on human chromosome 5q31.1 and named as TCF7 at the Human Gene Mapping 11 workshop [12]. TCF7 gene contains multiple exons, of which different splicings transcribe to variants of mRNAs. There are two promoters, four alternative exons (exon1a, 1b, 4a, and 9), and three splice acceptors in the exon 10 of TCF7 gene, while 96 different mRNAs could be theoretically translated [13]. At least 16 different protein isoforms with distinct functional properties have been found. mRNAs transcribed from the two different promoters can translate to two groups of proteins with different functions. The first promoter generates mRNA encoding a full-length activating form (FL-TCF7) (42–60 kDa), while the second intronic promoter produces a truncated, dominant-negative isoform of TCF7 (dnTCF7) (25–40 kDa) [14, 15]. The long isoforms contain a C-terminal DNA-binding domain named HMG box (encoded by exons 6 and 7), a Groucho binding domain, as well as an N-terminal β-catenin-binding domain (encoded by exon 1a and 1b), while the short isoforms lack of the β-catenin-binding domain [13, 16–18] (Fig. 1).

TCF7 was initially found to be expressed exclusively in T lineage lymphocytes [10], and the level of TCF7 was high in thymocyte cells and peripheral naive T cells, but was undetectable in effector T cells [13, 15, 19]. After then, TCF7 was also found in other tissues in embryonic period, mainly in neuroectodermal cells and differentiating mesenchymal cells. The expression of TCF7 seemed to be “shut off” around birth in non-lymphoid tissues [20], while TCF7 was over-expressed in malignant tumors, e.g. colorectal cancers [21], prostate cancers [22], or breast cancers [23], in addition to lymphomas and leukemia derived from T lymphocyte [19, 24]. However, a number of studies demonstrated that TCF7 might act as a tumor suppressor and be diminished in leukemia [25, 26], lymphomas [27], or colorectal cancers [28]. The dual roles of TCF7 in tumorogenesis could be explained by the different functions of variant isoforms of TCF7 and the complexity of tumor variations with multiple signaling pathways in the process of tumor development. The distribution of different TCF7 isoforms was found to vary between tumors and normal tissues in colon cancers or mammary tumors [29, 30]. It was detected that the dominant TCF7 isoform had short-life and existed in both nucleus and plasma in normal cells such as proliferating intestinal epithelial cells and basal epithelial cells of mammary gland epithelium, while the full-length isoform in tumor cells and mainly appeared in plasma. The balance between the full- and short-length isoforms seems to be a checkpoint for promoting or inhibiting tumor genesis and development.

#### Regulating and regulated roles of TCF7 gene

**Fig. 2:**
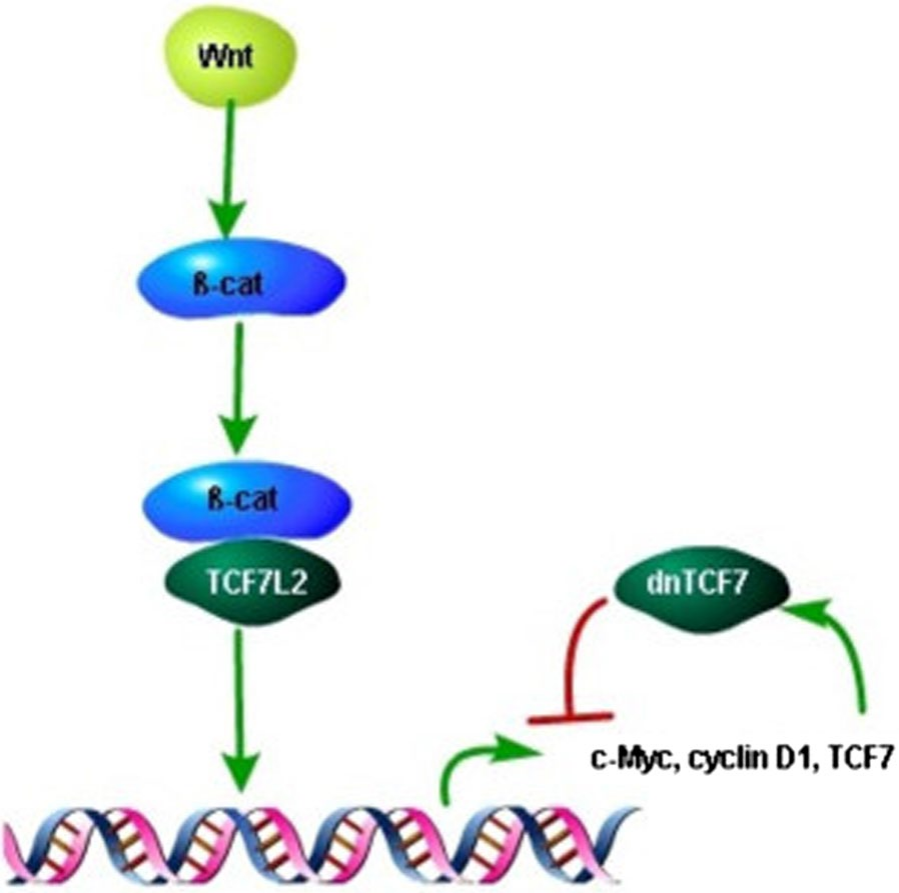
Negative feedback loop between β-catenin/TCF7L2 and TCF7. The Wnt signaling leads to the accumulation of β-catenin in the nucleus, which bind to TCF/LEF family of transcription factors. TCF7L2 is one of the TCF/LEF family members, which activates transcription of target genes together with the coactivator β-catenin. Although as a TCF/LEF member, TCF7 is also a target gene of β-catenin/TCF7L2. The dominant negative isoform of TCF7 protein negatively feedback to β-catenin/TCF7L2, functions as a transcription repressor, and down-regulates β-catenin/TCF7L2target genes. *β-cat* β-catenin, *TCF7L2* transcription factor 7-like 2, *dnTCF7* dominant negative transcription factor 7.

The expression of TCF7 gene is regulated by a number of factors through multiple signaling pathways. TCF7 is enhanced by β-catenin and TCF7L2 (also known as TCF4) as a downstream target gene of Wnt pathway. Two TCF-binding motifs were found at 1.2 kb upstream of promoter I of TCF7 gene on a CpG island that acts as an enhancer. The enhancer can be trans-activated by the combination of β-catenin and TCF7L2, while the dominant negative isoform of TCF7L2 could inhibit the transcription. TCF7 represses the target genes of β-catenin and TCF7L2, forming a negative feedback loop (Fig. 2). The process can be regulated through the counteraction of dnTCF7 with TCF7L2 target genes such as c-myc and cycline D1 [30]. TCF7 promoters do not contain the A/TA/TCANA G binding motif that can interact with TCF7 protein, therefore people used to consider that TCF7 could not be autoregulated [18]. But recent studies found that TCF7 could bind to the TCF7 locus and induce TCF7 gene expression [31].

TCF7 may be involved in the T cell differentiation, evidenced by the finding that TCF7 expression highly expressed in naïve T cells, down-regulated in effector T cells, and up-regulated again in memory T cells in the process of T cell differentiation [32]. TCF7 was proposed to regulate the activation of T cells and the production of their cytokines, and be simultaneously regulated by TCR stimulation and cytokines such as IL-4, IL-2, IL-15, IL-7 and IL-12 in human naïve CD8 T cells, especially through the dnTCF-1 isoform [15, 17, 33]. T cell cytokines could inhibit the inhibitory isoform of TCF7 and then facilitate the activation of naïve T cells into effector T cells, since TCF7 functions as a promoter factor in T cell differentiation.

#### Dual roles of TCF7 in transcription

**Fig. 3:**
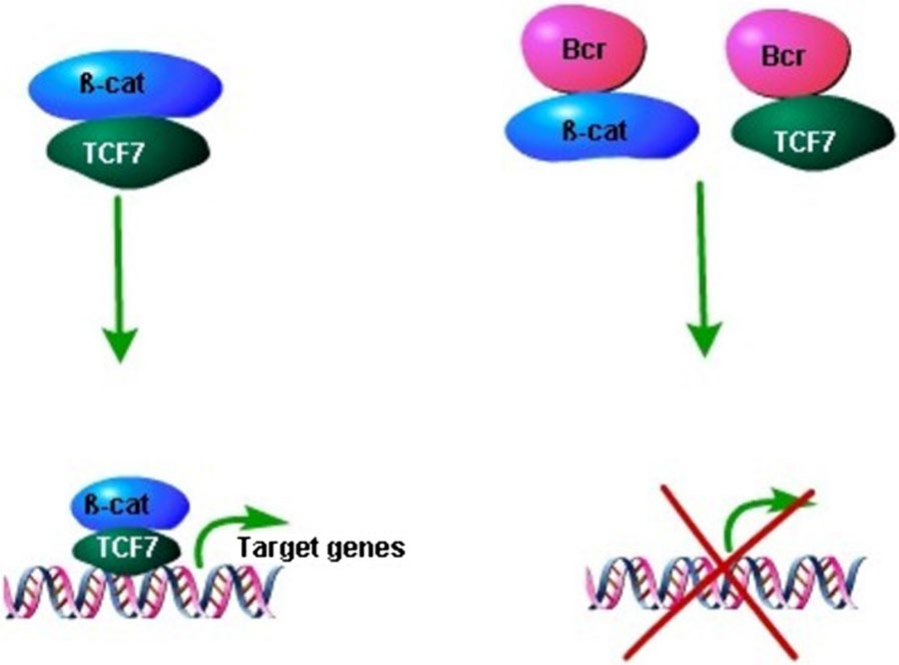
Bcr inhibits TCF7 mediated transcription. Bcr inhibits TCF7 mediated transcription through binding with both β-catenin and TCF7 protein, interferes the formation of β-catenin/TCF7 transcription complex, and therefore represses the target gene transcription. *β-cat* β-catenin, *TCF7* transcription factor 7, *Bcr* breakpoint cluster region.

TCF7 protein is generally known as a transcription factor. FL-TCF7 functions as a transcription activator, while dnTCF7 acts as a transcription repressor. Both isoforms of TCF7 could bind with Groucho co-repressors and act as a transcription repressor without β-catenin signaling, while FL-TCF7 could interact with β-catenin and induce the transcriptional activation when β-catenin accumulating in the nucleus. The dnTCF7 isoform lacks the N-terminal β-catenin binding domain and was proposed to play a negative role in transcription regulation [34]. The FL-TCF7 was considered to have dual functions in regulating gene transcription through the interaction with different proteins. In addition to Groucho family proteins, the β-catenin/TCF7-mediated transcription process could also be interfered directly by a multidomain protein, Bcr (breakpoint cluster region), to dissociate β-catenin/TCF7 complex and down-regulate the level of β-catenin/TCF7 target genes such c-Myc [35, 36], as shown in Fig. 3. Exogenous aptamers can also affect the formation of β-catenin/TCF7 complex, for example, RNA aptamer could specifically bind to TCF7 and inhibit its binding to β-catenin [37].

#### Role of TCF7 in the development of T cells and ILC2

**Fig. 4:**
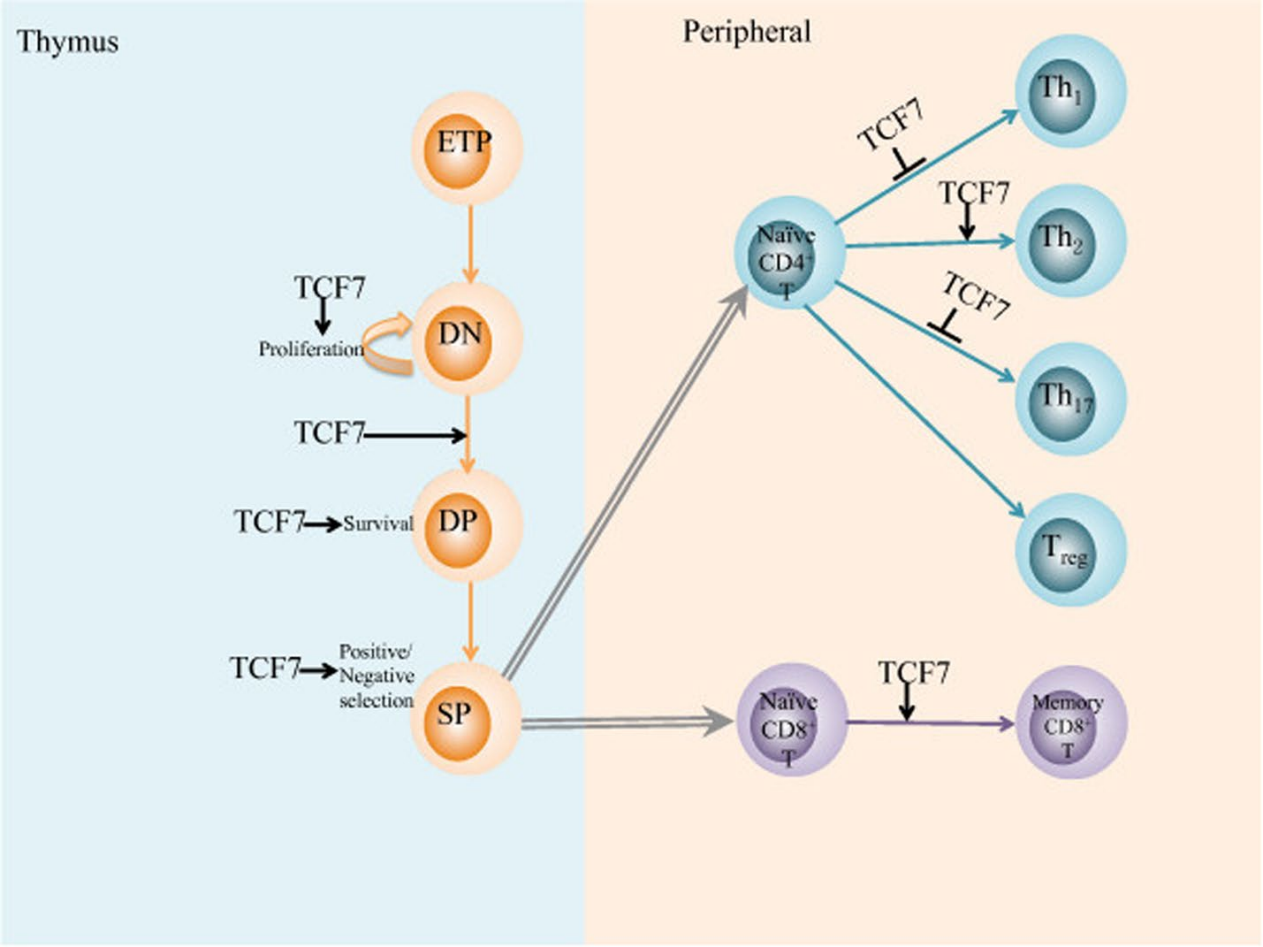
Role of TCF7 in T cell differentiation in thymus and peripheral. In thymus, early thymic progenitor (ETP) cells undergo several stages to form mature T cells. The process can be generally divided into three stages: double negative (DN), double positive (DP), and single positive (SP). TCF7 is involved in many critical events in the process, and is essential for DN cell proliferation, DN to DP transition, DP cell survival, the positive selection and negative selection. TCF7 is also important for peripheral T cell differentiation. TCF7 promotes CD4^+^ T cells differentiates to Th2 cells and suppress Th1 or Th17 cells at the same time. Besides, TCF7 also facilitates the formation of memory CD8^+^ T cells. *TCF7* transcription factor 7, *ETP* early thymic progenitor, *DN* double negative, *DP* double positive, *SP* single positive, *Treg* regulatory T cell.

TCF7 is an important factor for the development and differentiation of T-lineage cells, both in the thymus and the peripheral. Progenitor T cells in thymus undergo several stages and differentiated into naïve T cells and migrate to the peripheral. The peripheral naïve T cells then undergos another differentiation upon antigen encounter, and become effector T cells [38]. TCF7 is found highly up regulated in early thymic progenitors (ETPs), promotes the expression of T-lineage genes, and is critical for T cell specification. Although TCF7 usually promotes gene transcription with the co-activator β-catenin, evidence showed that the function of TCF7 in early T cell development is not relied on Wnt/β-catenin pathway [39, 40], but is closely associated with the activation of Notch signaling [31]. Even so, Wnt/β-catenin/TCF7 pathway is still involved in multiple stages of T cell development, supports proliferation of DN thymocytes, promotes DN to DP transition, enhances DP thymocyte survival and participates in positive/negative selection [38]. As in the peripheral, TCF7 was considered as a contributor for Th2 but suppressor for Th1 or Th17 cell fate in CD4+ T cells [17, 41–43], and also promotes the formation of memory cells in CD8+ T cells [44] (Fig. 4).

Group 2 innate lymphoid cells (ILC2) are a kind of innate lymphocytes that functionally produce Th2 cell associated cytokines and mediate innate type 2 immunity [45, 46]. A recent study demonstrated that TCF7 is required for the development of ILC2 cells, through both GATA-3-dependent and GATA- 3-independent pathways [47].

### Biological interactions with others

#### Interactions with CD3E

CD3E gene encodes CD3-ε polypeptide, which forms the T-cell receptor-CD3 complex together with CD3-γ, -δ and -ζ, and the T-cell receptor α/β and γ/δ heterodimers. TCF7 recognizes the motif sequenced as AACAAAG in the enhancer of CD3E gene, and induces the T-cell-specific expression of CD3-ε polypeptide. TCF7 was also found to be able to interact with TCR-α, TCR-β and TCR-δ enhancers. TCF7 could promote the formation of the TCR/CD3 complex, and act as a key factor in T cell development [10]. The low expression of CD3-ε was noted in both CD4+ T and CD8+ T cells from peripheral blood and pleural effusion of lung adenocarcinoma patients. Previous studies showed that NSCLC cells could induce the down-regulation of CD3-ε in Jurkat T cells which might be responsible for T-cell anergy in lung cancer [48]. It is possible that the down-regulation of CD3-ε in patients with lung cancer may be related to TCF7 dysfunction through the regulation of TCF7 in CD3E expression.

#### Interactions with β-catenin

**Fig. 5:**
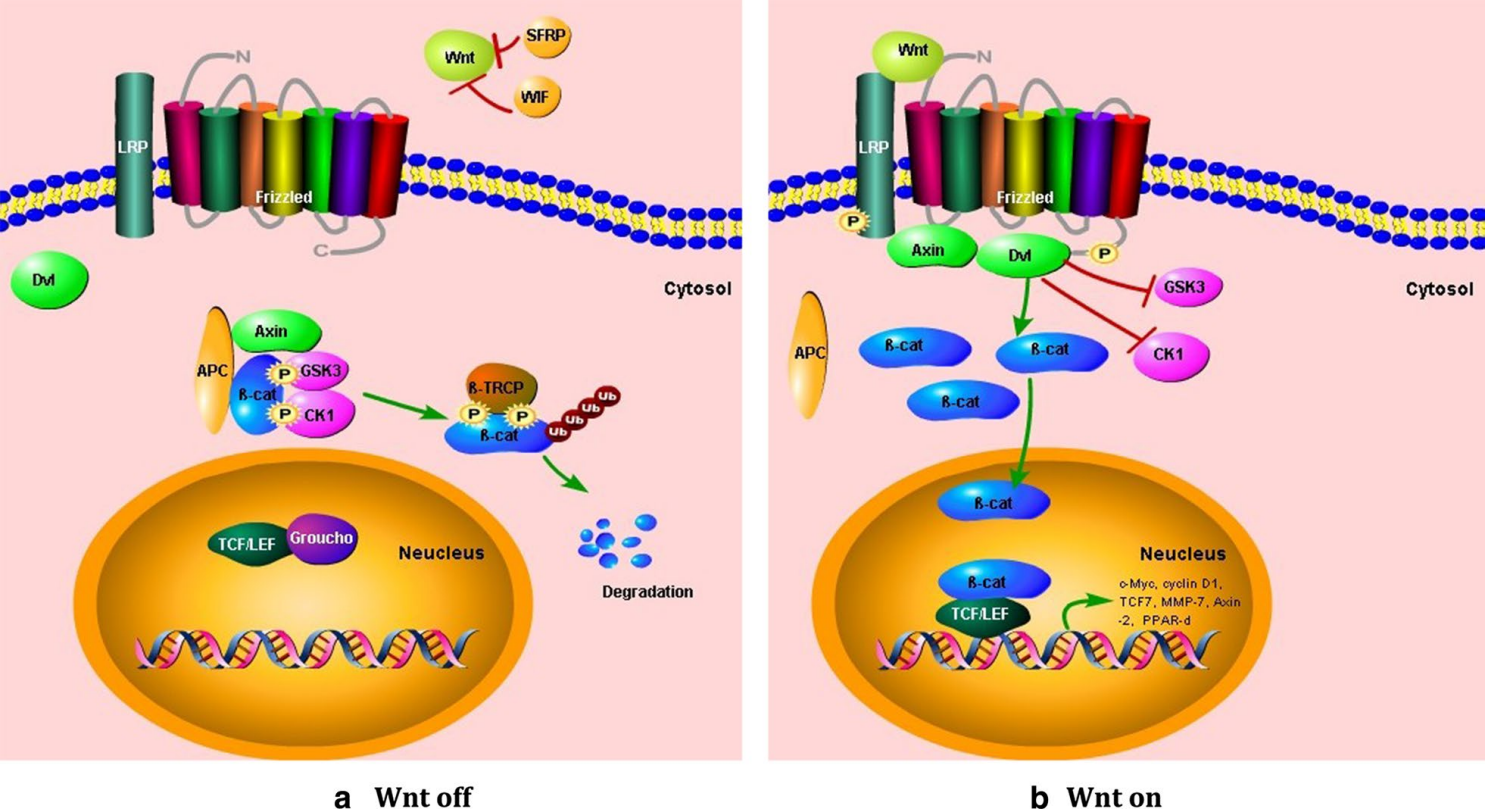
The canonical Wnt pathway. **a** Wnt is naturally inhibited by inhibitors such as SFRP and WIF. Without a Wnt signal, the β-catenin is captured by APC and Axin, then CK1 and GSK3 phosphorilate β-catenin, leading to proteasomal degradation. Without β-catenin binding, the TCF/LEF family proteins bind with Groucho family proteins, and function as a transcription inhibitor. **b** When Wnt signaling activated, Wnt binds to specific Frizzled-LRP complexes on the cell surface and initiate the canonical Wnt pathway. The activated receptor complex phosphorylates Dvl. Then Dvl inhibits CK1 and GSK-3, resulting in the accumulation of non-phosphorylated β-catenin in cytoplasm, which cannot be ubiquitinylated for degradation. β-catenin then translocates into the nucleus, and binds with TCF/LEF, causes transcriptional activation of target genes. *β-cat* β-catenin, *LRP* low-density lipoprotein receptor-related protein, *CK1* casein kinase 1α, *GSK-3* glycogen synthase kinase-3β, *Dvl* disheveled, *APC* adenomatous polyposis coli, *SFRP* secreted Frizzledrelated protein, *WIF* Wnt inhibitory factor, *TCF* T cell factor, *LEF* lymphoid enhancer-binding factor.

β-catenin, encoded by the CTNNB1 gene on human chromosome 3p21, is a key protein in canonical Wnt pathway [49], as shown in Fig. 5. Activation of Wnt signaling results in the accumulation and/or translocation of the downstream β-catenin in cytosol and/or nucleus, to form an active transcription complex with TCF/LEF family members including TCF7, TCF7L1, TCF7L2 and LEF1 [50]. β-catenin binds to TCF7 at the N-terminal β-catenin binding domain, which only exists in the full-length isoform of TCF7. The β-catenin/TCF7 signaling was reported as an important pathway in regulating T cell development, differentiation, or survival [38, 51]. TCF7 gene can encode a protein to form the transcription complex with β-catenin and act as a target gene of Wnt/β-catenin signaling, which could be enhanced by β-catenin/TCF7L2, together with Myc, Cyclin D1, PPAR-δ, MMP-7, Axin-2, or CD44, etc. [30, 50]. There might be a negative feedback loop whenTCF7 repressed β-catenin target genes [30].

#### Interactions with TCF7L2

TCF7L2, also known as TCF4, is another member of TCF family. There still remains a significant variation between TCF7L2 and TCF7, although both proteins function as transcription factors that bind to the coactivator β-catenin to up-regulate the transcription of target genes. The two proteins can interact with each other and form a regulation loop, as shown in Fig. 2. TCF7L2 binds to and transactivate the enhancer of TCF7 gene with the coactivator β-catenin and promotes TCF7 transcription, while TCF7 down-regulates TCF7L2 target genes and acts as a feedback repressor of β-catenin/TCF7L2 with a potential function of tumor suppressor [30]. High expression or mutations of TCF7L2 were found in several human tumors such as colorectal cancer [52], breast cancer [53], liver carcinoma [54], or lung cancer [55]. TCF7L2 was suggested to play an important role in the pathogenesis of NSCLC. However, a recent meta-analysis came to a contradictory conclusion that there is no association between TCF7L2 polymorphism with lung cancer [56]. The potential tumor-genesis role of TCF7L2 suggested a novel method in tumor therapies, which is to inhibit TCF7L2 expression or to down-regulate its target genes’ expression. Since TCF7 could suppress TCF7L2, it might be a promising anti-tumor strategy to introduce TCF7 to tumor cells as a treatment.

#### Interactions with LEF-1

Lymphoid enhancer-binding factor 1 (LEF-1), also named TCF-1a, is another TCF family member and contains a DNA-binding HMG box acting as a transcription factor, similar to TCF7. LEF-1 expresses in T cells and pre-B cells to promote thymocyte maturation as a collaborator of TCF7 [57, 58]. As downstream factors in wnt/β-catenin signaling, both TCF7 and LEF1 levels were up-regulated in some tumors such as colon cancers [59]. However, more recent studies found a restriction of LEF-1 in early thymocytes by the interaction with TCF7, so the malignant transformation of developing thymocytes could be prevented as a therapeutic target. LEF-1 is repressed directly by TCF7 through binding at TCF1 binding cluster (TBC), a cluster of three motifs located around −4.4 kb in the Lef1 locus [25].

#### Interactions with IL-17

IL-17, which is an important cytokine involved in infection, autoimmune response and allergy, is mainly produced by Th17 cells to promote the formation of Th17 cells [60]. A special link between IL-17 and asthma has been concerned, especially IL-17A and IL-17F of the IL-17 family, which were shown to play a pro-inflammatory role in asthma and have the potential association with the disease severity [61]. IL-17A was also suggested to be correlated with other airway diseases such as COPD [62]. It was found that TCF7 could repress the expression of IL-17 gene through the direct binding with the promoter region and the second intron of the IL-17 gene locus. Such repression was proposed to have no association with the other factors involved in Th17 differentiation, such as RORγt, Stat3, RORα, Ahr, Runx-1, Ets-1, Socs3, IRF4, or Batf. There was no connection with β-catenin either, although it acts as the co-activator of TCF-1 in many other cases [42]. Thus TCF7 could be a potential therapeutic target for asthma, COPD and other lung diseases associated with high-expression of IL-17 through inhibiting IL-17 expression.

#### Interactions with IL-4

**Fig. 6:**
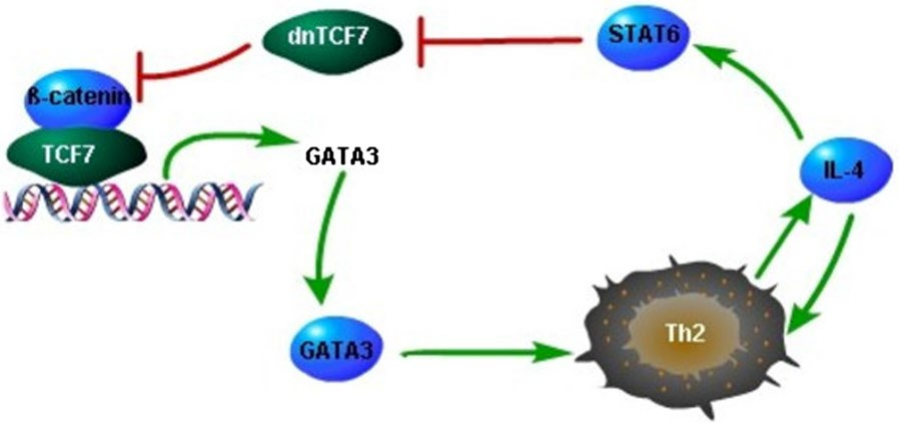
Interaction of TCF7 and IL-4. TCF7 promotes Th2 cell differentiation through transcriptional activation of GATA3. Th2 cells produce IL-4, which stimulates STAT6 and inhibits dominant negative TCF7, which represses the transcription of TCF7 target genes. Therefore, through inhibition of dn-TCF7, the process of Th2 cell differentiation is promoted. *TCF7* transcription factor 7, *dnTCF7* dominant negative transcription factor 7, *GATA3* GATA binding protein 3, *IL-4* interleukin 4, *STAT6* signal transducer and activator of transcription 6.

IL-4 is a main Th2 cytokine to promote the differentiation of naïve T cells to Th2 cells, produced by Th2 cells, subsequently [63]. IL-4 can stimulate B cell function [64] and is associated with chemotaxis of monocytes, macrophages and eosinophils [65, 66]. Paradoxically, anti-inflammatory functions of IL-4 was also found since it down-regulates TNF-α, IL-1, IL-6, IL-12, PGE2, or IL-8, which are proinflammatory mediators [67]. IL-4 displays the dual role in the course of lung fibrosis, plays an anti-inflammatory role in initial stages of lung injury, and promotes collagen deposition during the later stages [68]. TCF7 can promote Th2 cell differentiation through transcriptional activation of GATA-3, mainly from the proximal GATA-3 promoter at the upstream of exon 1b. IL-4 produced by Th2 cells down-regulates the expression of TCF7 gene and functions as a suppressor of TCF7 in naïve human CD4+ T cells, mainly through stimulation of STAT6 that interacts with specific DNA motifs of human TCF7 locus [17, 69]. IL-4 mainly down regulates the short isoform of TCF7, which functions as a transcription repressor and inhibit TCF7-mediated Th2 differentiation, thus contributes Th2 differentiation (Fig. 6) [17].

#### Interactions with Eomes

Eomesodermin (Eomes) is a transcriptional factor involved in CD8+ T cell memory [70]. TCF7 directly and specifically binds to 6 conserved consensus sequences in the Eomes 5′-regulatory region, to induce its expression and promote the formation and maintenance of memory CD8+ T cells [44]. Memory CD8+ T cells in airways are involved in the immediate immune response to secondary virus challenge, and provide a protection from secondary infection [71]. Introducing TCF7 to the lung would probably be a therapeutic way to protect patients from secondary infections through enhancing eomes.

### Involvements of signal pathways

#### Wnt signal pathway

The canonical Wnt pathway regulates a variety of cellular processes, including proliferation, differentiation, survival, apoptosis and cell motility, and plays an important role in lung morphogenesis, repair after injury, or carcinogenesis (Fig. 5) [72]. When Wnt pathway is activated, secreted signaling proteins of the Wnt family bind to specific Fzd-LRP (Frizzled-low-density lipoprotein receptor-related protein) receptor complexes on the cell surface and initiate the canonical Wnt pathway, leading to the intracellular accumulation of β-catenin [50]. The activated receptor complex phosphorylates cytoplasmic disheveled (Dvl) which then inhibits casein kinase 1α (CK1) and glycogen synthase kinase-3β (GSK-3). The activation of Dvl-CK1-GSK3 causes the failure of β-catenin degradation through ubiquitination and accumulation of non-phosphorylated β-catenin in cytoplasm. The stable β-catenin is then translocated into the nucleus to form an active transcription complex with TCF/LEF family members, resulting in activation of multiple target genes, such as c-myc, cyclin D1, matrix metalloproteinases (MMP2, MMP3, MMP7, and MMP9), Cox-2, c-jun, Fra-1, VEGFR, or TCF7 [73–82]. In the absence of Wnt ligand, TCF/LEF could repress the transcription of target genes through the interaction with Groucho family protein, and function as transcription repressors [34]. TCF7 also functions as a target gene of Wnt pathway. TCF7L2/β-catenin could activate the transcription of TCF7, while the dominant negative isoform of TCF7 repressed target genes of TCF7L2/β-catenin [30]. Therefore, different isoforms of TCF7 play totally different roles in the activation of Wnt signaling pathway.

The abnormality of Wnt signaling was proposed to be associated with the pathogenesis and development of lung diseases, e.g. cancer, fibrosis, or pulmonary arterial hypertension [79]. Activated Wnt signaling was found in NSCLC, with overexpression of Wnt proteins or Dvl, the lack of Wnt pathway repressors, e.g. WIF, sFRP1 and DKK3, or methylation of the promoter of APC, which binds and inhibits β-catenin [83–87]. Target genes such as LEF1 and HOXB9 were found to be related with the metastasis of lung adenocarcinoma [88]. The Wnt signaling might be also involved in the initial phase or in the ongoing multistep process of lung cancerogenesis [79]. However, the high level of β-catenin was found correlated with better prognosis in patients with NSCLC, which seems totally different from colon carcinomas or hepatomas [89–91]. The contradictory findings may be explained by the multi-function of β-catenin, which transduces canonical Wnt pathway and regulates cellular adhesion through the interaction with E-cadherin [91]. Overexpression of Wnt genes (WNT2 and −5a), the receptors (FZD7 and −10), WNT regulators (sFRP1 and −2), and Wnt target genes (MMP7) was reported in lung fibrosis [92–96], while the expression of TCF7 did not increase [97]. Wnt/β-catenin signaling was suggested involved in the repair process after lung injury, characterized by a decrease of inflammation, re-epithelialization, and matrix remodeling. Increased nuclear β-catenin was found during the fibroproliferative phase after acute lung injury. In addition to the Wnt/β-catenin signaling, the β-catenin/E-cadherin pathway was related with the repair process [98]. However, there were a number of contradictory reports that β-catenin was not necessary in the process of bronchiolar epithelium repair [99]. The paradoxical results may indicate the complexity of the disease pathology and the multiple roles of the Wnt pathway in lung diseases.

#### Activation in FoxO1 signal pathway

FOXO1 belongs to the forkhead family of transcription factors and contains a conserved forkhead domain which binds to specific DNA sequence and is involved in a number of cellular processes, including cell proliferation, apoptosis, differentiation, DNA damage or repair, and stress responses [100–102]. FOXO1 up-regulates p27 and p21 to down-regulate the cell cycle regulator cyclin D1, which results in the cell cycle arrest, and plays a potential role in tumor suppression [103]. FOXO1 expression was associated with an earlier stage of lung cancer, the less nodal involvement or venous invasion, or a favorable prognosis in NSCLC where apoptosis was induced by FOXO1 [104, 105].

FOXO1 plays an important role in the process of memory T cell differentiation. It activates the expression of TCF7, IL7r or Ccr7, through the direct interaction between the highly conserved forkhead-binding motif and the promoter region of the target genes [106]. FOXO1 could bind to multiple sites close to or within TCF7 gene of regulatory T cells and naive CD4+ T cells [107]. The activation of Akt phosphorylation could directly inactivate FOXO1 and down-regulate TCF7 expression [108].

#### The importance of Notch signal pathway

Notch pathway regulates critical cell fate decisions during both development and adult life [109], including the course of T cell development and differentiation, e.g. the decision of CD4+ T cells between the Th1 vs the Th2 fate [110]. TCF7 is one of the target genes of Notch to be up-regulated during the early development of T cells [31, 111]. Notch could directly activate TCF7 through the interaction with an enhancer 31.5 kb upstream of the TCF7 promoter, up-regulating the T-cell essential genes, including components of the TCR, or transcription factors Gata3 and Bcl11b [31]. Although TCF7 is the downstream of Notch, the consistent expression of TCF7, GATA3, or Bcl11b was independent on Notch, since TCF7 could positively auto-regulate and maintain the expression after the activation [31, 112]. It is probable that secondary factors other than Notch signaling were associated with the up-regulation of TCF7 and GATA3 expression during T cell differentiation stages of the DN-DP transition [112, 113]. GATA3 expression promotes the differentiation of T cells to Th2 cells or innate lymphoid cells (ILCs). It was reported that TCF7 with its coactivator β-catenin negatively regulated Notch pathway in thymocytes at CD4-CD8-double-negatve 3 (DN3) stage or beyond. The expression and signaling of pre-TCRα as Notch targets was repressed by TCF7 [114], although the mechanism by which TCF7 mediated the repression of Notch remains unclear.

#### The significance of P21 signal pathway

**Fig. 7:**
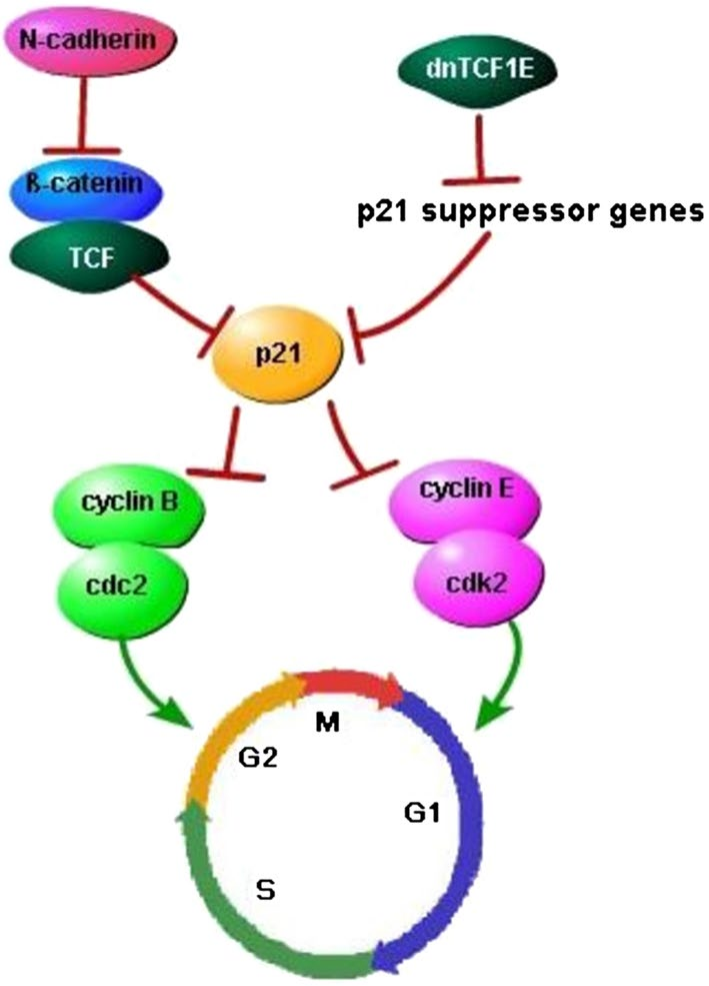
TCF7 in p21 pathway. Different isoforms of TCF7 plays totally different role in p21 pathway. The active TCF, includes the full-length TCF7 isoform, down-regulates p21, which may inhibits the cyclinB/cdc2 induced G2 to M phase transition. The β-catenin/TCF mediated cell cycle stall at G2 phase can be repressed by N-cadherin. Conversely, the negative isoform of TCF7, one of which is known as dnTCF1E, inhibits the p21 repressor genes, thus induces p21 expression, and arrests the cell cycle at G1 phase through inhibition of cyclinE/cdk2 complex. *TCF* T cell factor, *dnTCF1E* dominant negative E-tail isoform of TCF1.

P21 is an important cyclin-dependent kinase (cdk) inhibitor and represses the G1-to-S phase transition mainly by inhibiting the activity of the cyclin E–cdk2 complex. P21 is related to tumor differentiation and acts as a significant factor predicting the prognosis of patients with NSCLC [115, 116]. The growth arrest mediated by p21 was clarified to be dependent upon the activity of C-clamp, a DNA-binding domain other than HMG-box in the E-tail isoform of TCF1. HMG-box binds Wnt response elements (WREs), while the C-clamp contacts with 5′-RCCG-3′elements (where R is G or A) upstream or downstream of WREs. The C-clamp-RCCG interaction could induce the activation of p21 through the down-regulation of multiple p21 suppressor genes like RUNX1, SMARCA4, SP5, TGIF, or YAP1 at the transcription level, MSI2 at the RNA stability, or CUL4A at the protein stability. The up-regulation of p21 by dnTCF1E may subsequently cause a stall in the G1 phase of the cell cycle [117]. Other studies found that the p21 promoter could be inhibited by constitutively active TCF, and enhanced by dominant negative TCF. N-cadherin suppressed β-catenin/TCF, induced p21 expression, and lead to a decreased activity of cyclinB-Cdc2 kinase and G2/M arrest [118] (Fig. 7).

### Potential roles in the pathogenesis of lung diseases

#### Pulmonary infection

TCF7 is involved in the development or differentiation of T cells in the thymus or peripheral circulation, promotes the naïve CD4+ T cells into Th2 cells and accelerates the formation of memory T cells, which may provide the protective roles in pulmonary infection [38]. TCF7 was also associated with development or differentiation of natural killer cells or ILC2 cells [47]. There is a need of the direct evidence to show the involvement of TCF7 in the development of pulmonary infection, even though TCF7 may have the close relationship with key lymphocytes in immune responses against infection.

#### Pulmonary inflammation

TCF7 was found differentially expressed upon airway inflammation. Gene microarray showed TCF7 significantly down-regulated in CD4+ T cells from the inflamed lung of SPC-HA/TCR-HA mice compared with the lung of healthy TCR-HA donors, while no expression difference was detected in CD4+ T cells from the spleen of the same mice [119]. It seemed to be a potential relationship between TCF7 and airway inflammation, however, the research did not clarify the different isoforms of TCF7, and did not show the expression difference between the FL-TCF7 that promotes Th2 response and dn-TCF7 that repressed by Th2-polarized lymphocytes. Both Th2 and ILC2 cells are lymphocytes associated with type 2 immunity, which is characterized by high antibody titers and is responsible for the development of asthma and other allergic inflammatory diseases through releasing Th2-type cytokines. Th2-polarized cells down-regulates the expression of dn-TCF7. Therefore, dn-TCF7 could probably be selected as a biomarker for airway inflammation [120]. Since FL-TCF7 promotes type 2 immunity, it could be a candidate gene target for anti-inflammatory therapy.

#### Allergy and asthma

TCF7 resides on human chromosome 5q31.1, where was proposed as a candidate loci associated with asthma and allergy through genome-wide screens [121]. Both Th2 cells and ILC2 cells promoted by TCF7 were found to be involved in asthma. An experimental model of allergic asthma induced by ovalbumin in mice showed TCF7 was required in the production of the Th2 cytokine, IL-4. The TCF7-deficient mice produce less IL-4 and showed less inflammation around airways, while the normal mice showed more inflammatory cell infiltration in perivascular and peribronchial areas after the challenge with ovalbumin. A diminished GATA3-1b expression was also detected in TCF7-deficient mice, suggesting that TCF7 induce IL-4 through GATA-3 pathway and contribute the development of airway inflammation [41]. ILC2 induced by TCF7 also contributes to Th2 response, and is closely related to asthma. A recent study found that during experimental asthma, ILC2 proliferate and produce IL-13, which contributes to allergy and worsen the condition of asthma [122]. The deficiency of TCF7 will lead to the lack of ILC2, and contribute relieving type 2 inflammations. Therefore, inhibition of TCF7 in the airway may play a protective role in allergic asthma, and might be considered as a promising target for the future treatment of asthma.

#### Acute lung injury

TCF7 expression was up-regulated in mice models of acute lung injury, accompanied by the increased β-catenin protein levels in nuclear localization [98]. β-catenin was suggested to play an important role in the repair phase after lung injury. The fibroproliferative repair was initiated and regulated by the non-canonical E-cadherin-β-catenin axis after lung injury induced by butylated hydroxytoluene/hyperoxia, through the regulation of the epithelial proliferation and lung matrix remodeling. The cytoplasma levels of E-cadherin decreased and the nuclear levels of β-catenin increased with an increasing expression of the cotranscriptional regulators, e.g. TCF7, TCF7L1, or target genes including cyclin D1 6 days after the induction of acute lung injury [98]. The expression of β-catenin increased in type II alveolar epithelial cells in animals with acute lung injury [123], probably different from the mechanism by which β-catenin was found to be involved in the repair and remodeling processes in patients with idiopathic pulmonary fibrosis [124]. In addition to the involvement of E-cadherin, the Wnt pathway was proposed as one of the signaling pathways involved in the repair phase after lung injury, evidenced by the finding that Wnt regulated lung morphogenesis and development [125]. However, it was found that the β-catenin was not necessary for the maintenance or efficient repair of the bronchiolar epithelium, since the knockout of β-catenin failed to affect the repair of the naphthalene-injured airway [99]. It indicates that other pathways may be involved in the repair process after lung injury, which should be furthermore clarified in future studies.

#### COPD/emphysema

COPD is the third leading cause of death worldwide, characterized by irreversible airflow obstruction and loss of functional pulmonary tissue [1]. Emphysema is the main feature of COPD with alveolar airspace enlargement, parenchymal tissue destruction, and impaired pulmonary regeneration [126]. Wnt/β-catenin signaling was found to be related with lung development and repair after lung injury and was proposed as a pathway associated with emphysema. The activity of the Wnt/β-catenin signaling pathway and the expression of its target genes, such as genes of TCF/LEF family, were observed decreased in lung tissues of patients with COPD or in experimental emphysema, where TCF7 even was not expressed [127, 128]. Increased activation of Wnt/β-catenin showed therapeutic effects in experimental emphysema by increasing pulmonary repair and decreasing airspace enlargement, attenuated the compromise of parenchymal tissues and restored the structure and function of alveolar epithelial cells [127]. These studies indicated that the up-regulation of the Wnt signaling pathway might be a therapeutic strategy for emphysema.

#### Lung cancer

TCF/β-catenin-mediated transcription is an important regulator for carcinogenesis of diverse cells [37]. Among target genes of TCF/LEF family, cyclin D1 and c-*myc* are responsible for the decision between proliferation and apoptosis in the cells, and matrix metalloproteinase-7 (MMP-7) is related with tumor metastasis [80, 129]. Wnt/TCF pathway activation was clarified closely associated with the tumorogenesis, development and metastasis of lung cancer, and the metastatic capacity of lung adenocarcinoma cells could be suppressed by treatment with dominant negative mutants of TCF7 and TCF7L2, which suggested that the negative isoforms of TCF7 and TCF7L2 might be potential therapies for lung cancer [88].

## Conclusions

TCF7 is one of important transcription factors for T cell development and differentiation, embryonic development, or tumorogenesis. Multiple TCF7 isoforms can be broadly divided into two groups: the full-length isoforms (FL-TCF7) as transcription activators, or dominant negative isoforms (dn-TCF7) as transcription repressors. TCF7 interacts with multiple proteins and target genes (e.g. CD3E, β-catenin, TCF7L2, LEF-1, IL-17, IL-4, or Eomes) and is involved in several signal pathways (e.g. Wnt, FoxO1, Notch, or P21pathways) which are critical for lung diseases, especially the canonical Wnt/β-catenin pathway. TCF7 is suggested to be involved in immune responses to pathogens, autoimmune diseases, pulmonary infection, allergy, or asthma through promoting Th2 response, or the formation of memory CD8+ T cells. TCF7 also plays an important role in tissue repair and remodeling after acute lung injury or in the development of pulmonary fibrosis. The dual roles of TCF7 in lung cancers were discussed. FL-TCF7 is associated with the proliferation, invasion, or metastasis of lung cancer cells and the poor prognosis of patients with lung cancers, while dn-TCF7 shows therapeutic effect for cancer metastasis. Thus, TCF7 and TCF7-associated regulations play critical roles in the pathogenesis and development of lung diseases and should be considered as a new therapeutic target.

## Abbreviations

Bcr: breakpoint cluster region
cdk: cyclin-dependent kinase
CK1: casein kinase 1α
COPD: chronic obstructive pulmonary disease
dnTCF1E: dominant negative E-tail isoform of TCF1
dnTCF7: dominant-negative isoform of TCF7
Dvl: disheveled
ETP: early thymic progenitor
Eomes: eomesodermin
FL-TCF7: full length isoform of TCF7
Fzd-LRP: frizzled-low-density lipoprotein receptor-related protein
GATA3: GATA binding protein 3
GSK-3: glycogen synthase kinase-3β
HMG: high mobility group
IL: interleukin
ILC: innate lymphoid cell
LEF: lymphoid enhancer-binding factor
LEF-1: lymphoid enhancer-binding factor 1
MMP-7: matrix metalloproteinase-7
SFRP: secreted Frizzledrelated protein
STAT6: signal transducer and activator of transcription 6
TBC: TCF1 binding cluster
TCF-1: T cell-specific transcription factor-1
TCF7: transcription factor 7
TCF7L1: transcription factor 7-like 1
TCF7L2: transcription factor 7-like 2
WIF: Wnt inhibitory factor
WRE: Wnt response element
